# Discovering Time-Varying Public Interest for COVID-19 Case Prediction in South Korea Using Search Engine Queries: Infodemiology Study

**DOI:** 10.2196/63476

**Published:** 2024-12-16

**Authors:** Seong-Ho Ahn, Kwangil Yim, Hyun-Sik Won, Kang-Min Kim, Dong-Hwa Jeong

**Affiliations:** 1 Department of Artificial Intelligence The Catholic University of Korea Bucheon-Si Republic of Korea; 2 Department of Hospital Pathology College of Medicine The Catholic University of Korea Seoul Republic of Korea; 3 Department of Data Science The Catholic University of Korea Bucheon-Si Republic of Korea; 4 Department of Healthcare and Aritifical Intelligence The Catholic University of Korea Bucheon-Si Republic of Korea

**Keywords:** COVID-19, confirmed case prediction, search engine queries, query expansion, word embedding, public health, case prediction, South Korea, search engine, infodemiology, infodemiology study, policy, lifestyle, machine learning, machine learning techniques, utilization, temporal variation, novel framework, temporal, web-based search, temporal semantics, prediction model, model

## Abstract

**Background:**

The number of confirmed COVID-19 cases is a crucial indicator of policies and lifestyles. Previous studies have attempted to forecast cases using machine learning techniques that use a previous number of case counts and search engine queries predetermined by experts. However, they have limitations in reflecting temporal variations in queries associated with pandemic dynamics.

**Objective:**

This study aims to propose a novel framework to extract keywords highly associated with COVID-19, considering their temporal occurrence. We aim to extract relevant keywords based on pandemic variations using query expansion. Additionally, we examine time-delayed web-based search behavior related to public interest in COVID-19 and adjust for better prediction performance.

**Methods:**

To capture temporal semantics regarding COVID-19, word embedding models were trained on a news corpus, and the top 100 words related to “Corona” were extracted over 4-month windows. Time-lagged cross-correlation was applied to select optimal time lags correlated to confirmed cases from the expanded queries. Subsequently, ElasticNet regression models were trained after reducing the feature dimensions using principal component analysis of the time-lagged features to predict future daily case counts.

**Results:**

Our approach successfully extracted relevant keywords depending on the pandemic phase, encompassing keywords directly related to COVID-19, such as its symptoms, and its societal impact. Specifically, during the first outbreak, keywords directly linked to COVID-19 and past infectious disease outbreaks similar to those of COVID-19 exhibited a high positive correlation. In the second phase of the pandemic, as community infections emerged, keywords related to the government’s pandemic control policies were frequently observed with a high positive correlation. In the third phase of the pandemic, during the delta variant outbreak, keywords such as “economic crisis” and “anxiety” appeared, reflecting public fatigue. Consequently, prediction models trained by the extracted queries over 4-month windows outperformed previous methods for most predictions 1-14 days ahead. Notably, our approach showed significantly higher Pearson correlation coefficients than models based solely on the number of past cases for predictions 9-11 days ahead (*P*=.02, *P*<.01, and *P*<.01), in contrast to heuristic- and symptom-based query sets.

**Conclusions:**

This study proposes a novel COVID-19 case-prediction model that automatically extracts relevant queries over time using word embedding. The model outperformed previous methods that relied on static symptom-based or heuristic queries, even without prior expert knowledge. The results demonstrate the capability of our approach to track temporal shifts in public interest regarding changes in the pandemic.

## Introduction

The COVID-19 pandemic caused by the SARS-CoV-2 virus has highly affected the clinical system as well as social systems worldwide. COVID-19 is highly contagious and can be lethal to some individuals, especially older adults, those with underlying medical conditions, and immunocompromised individuals. Owing to the rapid global spread of COVID-19 in March 2020, 3 months after the first case was reported in December 2019 in Wuhan, China, the World Health Organization declared the outbreak a public health emergency of international concern. Most countries use the daily number of confirmed COVID-19 cases as a key indicator for determining quarantine policies to prevent its spread. These quarantine policies impact people’s daily lives and economic activities, such as increasing delivery orders for food, allowing telemedicine, and enabling teleworking. Therefore, the daily number of COVID-19 confirmed cases has become a significant barometer for guiding policies and lifestyle changes during the post–COVID-19 era.

Numerous attempts have been made across several countries to forecast the number of confirmed COVID-19 cases using machine learning techniques [1-12]. Most studies have used a previous number of cases to capture the temporal dynamics of time-series data using autoregressive [2,3], conventional machine learning [4-6], and deep learning [7,8] models. However, using only the previous number of cases cannot reflect temporal variation due to external factors such as outbreaks with social events, new variants, and quarantine policy. To mitigate this limitation, some studies used additional features including traffic [9,10], mobile roaming [11], and search engine query [12] data. These approaches have demonstrated improvements in forecasting performance by reflecting human behavior and interest during the COVID-19 pandemic.

Web-based behaviors such as searching for and communicating on social networking services are significantly associated with the spread of infectious diseases [12-14]. In particular, the frequency of search engine queries has proven to be one of the most important complementary datasets for predicting infectious diseases such as COVID-19 [12,15-18]. Specifically, previous studies [15-18] demonstrated the feasibility of accurately predicting the number of confirmed COVID-19 cases by using search engine query data. A previous study [15] demonstrated that models incorporating search engine queries related to COVID-19 symptoms exhibited improved forecasting performance compared with those that did not include such queries. In these studies, query selection was important for extracting useful search engine queries that correlated with the confirmed cases. Previous studies [15-18] were selected based on correlation studies on medical expertise and COVID-19–related symptoms. However, because some studies [15-18] selected queries based on heuristic human knowledge (ie, selection by expertise knowledge), choosing relevant keywords demands a high level of expertise and continuous updating as time progresses. Consequently, previous studies may exhibit limitations in accurately reflecting real-world situations, as they did not consider the temporal fluctuations of search terms associated with COVID-19. To address this issue, it is essential to automatically extract relevant queries by considering the search behavior across different phases of the pandemic. We applied query expansion using word embedding to develop an automatic query extractor. Word embedding enables the understanding of word meanings by analyzing contextual word occurrences and empowering the automatic extraction of strong relationships among COVID-19–related terms in a corpus.

In this study, we propose a novel framework to extract terms strongly associated with COVID-19 considering their temporal occurrence. We used query expansion using word embedding to overcome the limitations of previous heuristic studies. From 8 major media outlets in South Korea, we collected news articles containing the word “COVID” by period. Then we trained word embedding models with the collected articles and selected the top 100 words with a high word similarity with “COVID” by period. We used these words to obtain expanded search engine query data. Additionally, we propose a time-lagged cross-correlation (TLCC) based feature selection method that considers the time delay between search engine queries and the number of confirmed cases. Subsequently, these terms were used as extended queries with an optimal time lag within the search engine data to predict the number of confirmed COVID-19 cases 1-14 days ahead.

In summary, our contributions are three-fold: (1) we developed a novel framework for daily COVID-19 case prediction by extracting efficient queries for query expansion by considering the temporal importance of queries, (2) the proposed query expansion can discover efficient queries for daily COVID-19 case predictions by capturing the transition of query trends over time, and (3) our framework can predict important keywords that change over time in real-world use by significantly outperforming the previous method of using search engine query data 1-14 days ahead of prediction.

## Methods

### Ethical Considerations

No ethics approval was required for this study, as it was not considered human participant research. All data were collected from publicly available sources. Data on confirmed COVID-19 cases were obtained from Our World in Data [19]. Search engine queries were collected from NAVER Data Lab (NDL) [20], one of the largest search engines in Korea.

### Our Framework

#### Overview

**Figure 1:**
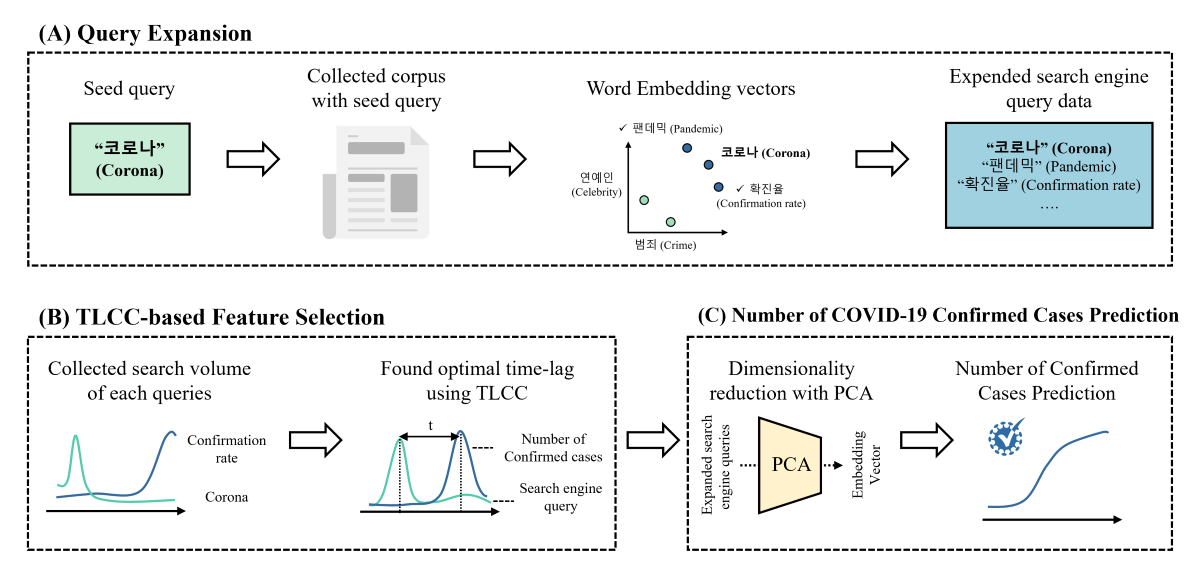
Overview of our framework. The framework comprises 3 modules: query expansion, feature selection, and prediction model. PCA: principal component analysis; TLCC: time-lagged cross-correlation.

Our framework consists of 3 components: query expansion, TLCC-based feature selection, and daily number of confirmed case predictions. Through query expansion, we performed word embedding of a COVID-19–related corpus collected beforehand, segmented by time period, followed by the extraction of word vectors. We then calculated the cosine similarity between these word vectors and the COVID-19–related terms to extract COVID-19–related queries. The extracted queries were used to gather search engine query data. By using the TLCC-based feature selection, we reflected on the time delay between search engine queries and the number of confirmed cases to determine the optimal time lag. Using the obtained optimal time lag, we conducted daily confirmed case predictions using expanded search engine queries. An overview of the proposed framework is presented in Figure 1.

#### Query Expansion

Query expansion is a method aimed at enhancing search efficiency by identifying multiple words related to the seed query. Various approaches apply query expansion methods that consider numerous words similar to the seed queries. The first automatic query expansion method applied using a statistical approach was discussed in a previous study [21]. However, this approach has limitations, because it inadequately considers the semantic features between words. Word embedding, a technique for learning vector representations of word semantics, has been extensively studied in natural language processing. Word2Vec [22] is one of the most widely used word-embedding methods. It vectorizes the meaning of a word by considering the contextual meaning of the sentence and uses a shallow neural network to match the words appearing around it.

Previous studies [23,24] have demonstrated the applicability of word-embedding features to query expansion for generating more relevant keywords. A previous study [23] applied the Word2Vec model to word embedding for query expansion. Another study [24] proposed a method that considers topic-specific words in query expansion using Word2Vec. While previous methods [23] have focused on the global meaning of words, this approach [24] considers the local meaning of words based on domain knowledge. For example, in the previous method [23], terms closely associated with “gasoline tax,” such as “cutting,” “squeeze,” and “reduce,” are generated, capturing the global context of the topic. In contrast, the approach proposed in [24] generates terms like “tax,” “deficit,” and “vote,” emphasizing a more localized semantic interpretation of domain knowledge.

Therefore, we applied this study’s concept to COVID-19–specific words. This approach aids in extracting specific keywords related to COVID-19 and automatically extracts queries from valuable search engine query data to predict daily COVID-19 cases. We trained Word2Vec on a collected COVID-19–specific corpus, extracting similar words between “코로나” (translated as “Corona”) and others using cosine similarity on the word vectors.

However, search engine query data are primarily driven by people’s interests, which can change over time, leading to shifts in the perceived importance of keywords. Previous methods have struggled to account for this temporal variation in semantics. To address this, we used a simple approach to transform data, considering the temporal variation in semantics over a window of 4 months based on the article publication date. We applied word embedding to each article with a striding 1-month interval to evaluate the model’s performance throughout the entire period. This method not only accommodates temporal changes in semantics but also conserves storage space, requiring relatively small amounts of training data in real-world use environments and minimizing the training time. We excluded single-letter words and extracted the top 100 words with the highest cosine similarity because single-letter words are likely to be meaningless. We extracted search engine query data using queries obtained by query expansion including “코로나 (Corona).”

#### TLCC-Based Feature Selection

In previous studies [25-27], search engine queries were shown to exhibit a time lag, occurring earlier than the confirmation of cases. Because query expansion involves the extraction of various keywords, each feature has a critical time lag for prediction. Consequently, we propose a feature selection method based on the TLCC. TLCC is a method that considers time lag, facilitating the measurement of similarity between 2 signals. This method enables the extraction of features with the maximum Pearson correlation coefficient (PCC) between search engine queries and the number of confirmed cases at the optimal time lag. We calculated the TLCC up to 14 days prior to the present day for search engine queries extracted through query expansion and the number of confirmed cases. The equations for TLCC-based feature selection are denoted as Equations 1, 2, and 3:




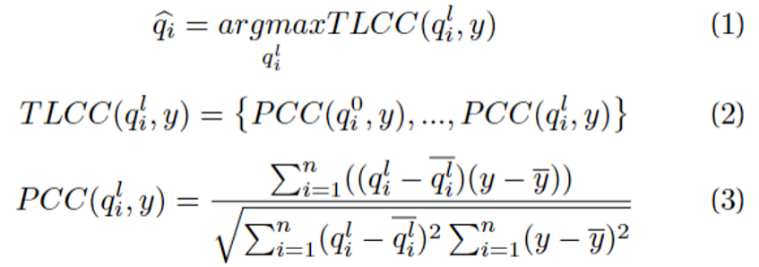




where *q* is a set of search engine queries; *i* is an index of each search engine query; and *l* is the range of the time lag, which is a hyperparameter for the TLCC and is the search engine query selected by the TLCC-based feature selection. We used the range of *l* to –14 ≤ *l* ≤ 0.

#### Prediction of the Number of Confirmed COVID-19 Cases

After extracting 101 words related to “코로나 (Corona)” through query expansion, we obtained search engine query data. Using the NDL application programming interface (API), we collected data for both the extracted words and “코로나 (Corona)” over a time series spanning 4 months. Within this period, we designated the last 30 days as the test set, and the preceding days as the training set. Using feature selection based on the TLCC, we applied it to each of the 100 keywords extracted through query expansion to extract features with an optimal time lag in the training set.

Not all 100 features automatically extracted through query expansion contribute effectively to the prediction; we used principal component analysis [28] to filter and retain only pertinent information for prediction. We reduced the dimensionality of the features using principal component analysis, with an explained variance of 0.90. Additionally, following the approach of previous studies [17], we used the cumulative number of confirmed cases in the past 3 days as an additional feature.

For data normalization, we applied a min-max scaler. To predict daily COVID-19 cases using search engine query data, we used ElasticNet [29], a linear regression model with L1 and L2 regularizations. ElasticNet is commonly used for the time-series prediction of high-dimensional data. It has been applied to predict daily COVID-19 cases in a previous study [18]. We selected the L1 and L2 regularization hyperparameters through 5-fold cross-validation using the prequential approach, considering the characteristics of the time series [30].

### Dataset

#### Daily Confirmed COVID-19 Case Data and Search Engine Query Data in South Korea

Daily COVID-19 case data represent the number of patients diagnosed with COVID-19 per day. This data was sourced from Our World in Data [19]. We used this dataset, covering the period from February 1, 2020, to November 30, 2021, for South Korea. The search engine query data represent the frequency of a search term on a search engine portal site per day. This study uses NDL API and Google Trends (GT), using queries extracted through the query expansion method, to predict confirmed COVID-19 cases in South Korea. We primarily conducted correlation analysis using NDL data, while the GT data were used for a comparative analysis of prediction performance against the NDL data. Data were collected for each of the 4 months, spanning from February 1, 2020, to November 30, 2021, considering various options such as age, sex, and device type. These values were normalized and ranged from 0 to 100, representing the actual search frequency within the user-defined period.

#### COVID-19–Specific Corpus Collection

Our focus was to build a corpus of texts highly relevant to COVID-19 to extract queries from search engine query data to predict daily COVID-19 cases. News articles from major media outlets tend to be more reliable than community posts and provide valuable information related to COVID-19; therefore, we collected news articles for query expansion. A COVID-19–specific corpus was amassed from news articles obtained from 8 news agency websites [31-38].

To mitigate the potential impact of political bias on keyword extraction, we balanced the selection of media outlets based on political learning. A total of 3 conservative, 3 liberal, and 2 centrist outlets were considered. To ensure the retrieval of exclusively COVID-19–related news articles, we crawled articles identified in search results using keywords such as “코로나19 (Corona19),” “코로나 (Corona),” “코로나 바이러스 (Coronavirus),” “신종 코로나바이러스 (Novel Coronavirus),” “COVID-19,” and “코비드19 (COVID-19)” from February 2020 to December 2021, leveraging BIG KINDS [39], a news big data analysis tool. Basic information regarding the collected text data is summarized in Multimedia Appendix 1. To extract meaningful Korean words, we removed all non-Korean languages and special characters. To extract nouns from the corpus for training word embeddings, we used an Open Korean Text tokenizer [40], a Korean language tokenizer. Open Korean Text tokenizer is trained on Twitter data and exhibits robustness to out-of-vocabulary issues, allowing it to distinguish COVID-19–related words, including neologisms such as “트윈데믹 (Twindemic).”

### Model Evaluation

In the evaluation, we used the mean absolute percentage error (MAPE), root-mean-squared percentage error (RMSPE), and PCC to measure the performance of the time-series prediction. Previous studies [41,42] used the mean absolute error (MAE), root-mean-squared error (RMSE), and PCC. Our aim was for the model to perform consistently well across all 15 periods. However, owing to the dependence of the MAE and RMSE on the scale of the ground truth, it is challenging to consistently measure the model’s performance across all periods in a consistent manner. Hence, we used MAPE and RMSPE as the percentage versions of MAE and RMSE, respectively. The MAPE and RMSPE quantify the disparity between the actual and predicted values as percentage values, with lower values being preferable. In contrast, PCC gauges the statistical resemblance between the actual and predicted values, with higher values being preferable. We compared the performance of our method with that of previous methods by averaging the evaluation metrics across all 15 periods. The equation of these evaluation metrics is denoted as Equations 4, 5, and 6.




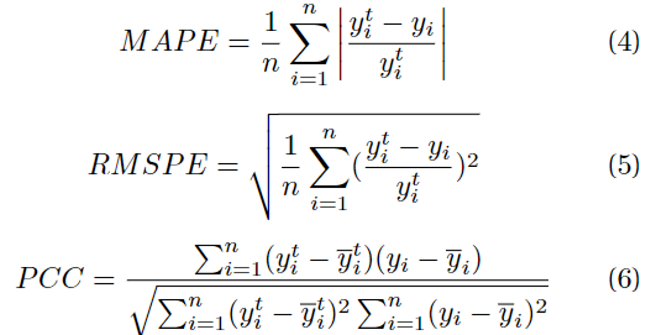




Where *y^t^* is the ground truth, 
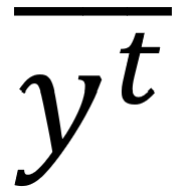
 is the mean of the ground truth, *y* is the predicted value, and 
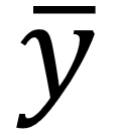
 is the mean of the predicted value.

In addition, we performed the Kruskal-Wallis H-test to compare our method with previous methods for multiple comparisons. We performed the Mann-Whitney *U* test for paired comparisons with False Discovery Rate Correction. This statistical analysis demonstrated that our method significantly outperformed previous methods in a statistically meaningful manner.

## Results

### Qualitative Analysis of Search Engine Query Data

**Figure 2:**
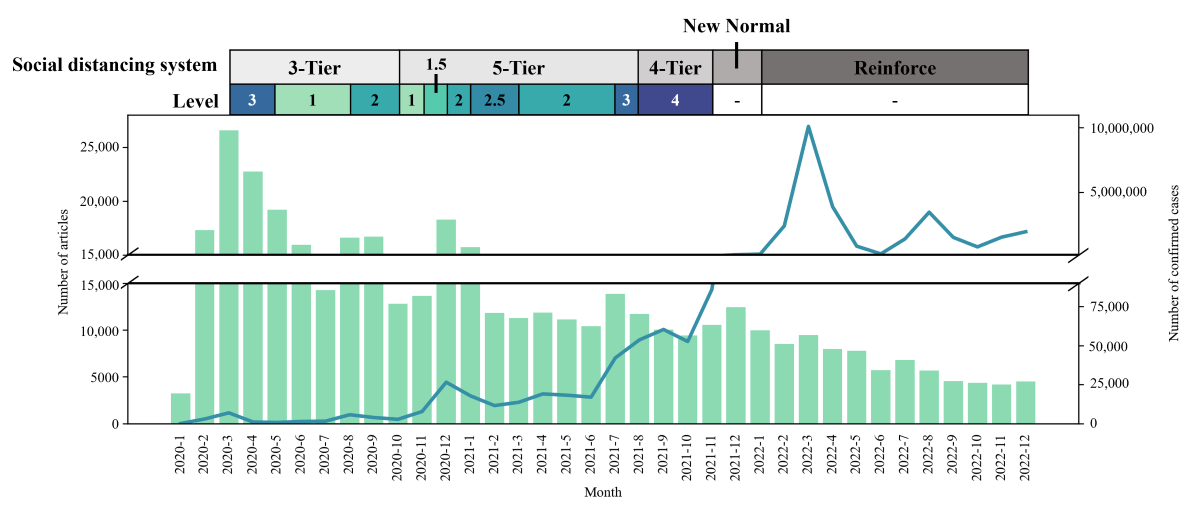
Number of articles and number of COVID-19 confirmed cases. The bar plot denotes the number of news articles related to COVID-19, while the blue line indicates the number of COVID-19 confirmed cases. The table at the top shows the social distancing system and levels announced by the Korean government.

We used extracted search engine query data based on the cosine similarity of word vectors correlated to “코로나” (translated as “Corona”) which is a Korean word on subword of “코로나바이러스-19” (translated as “Coronavirus-19”). Therefore, it was necessary to collect a sufficient number of news articles to extract useful word vectors. Figure 2 demonstrates the number of confirmed COVID-19 cases along with the number of news articles when searching articles with the keywords “코로나19 (Corona 19),” “코로나 (Corona),” “코로나바이러스 (Coronavirus),” “신종 코로나바이러스 (Novel Coronavirus),” “COVID-19,” and “코비드19 (Covid19),” which are paraphrased Korean word of “COVID-19.” After the first case occurred on January 20, the number of articles changed over the following months. The highest number of news articles reported group infections caused by Shincheonji in March 2020. As the number of confirmed cases decreased, the number of news articles also declined gradually. In December 2020, with the resurgence of COVID-19, and in July 2021, with the increase in confirmed cases due to the delta variant, the number of news articles increased. However, following the emergence of the omicron variant in December 2021, despite a rapid increase in confirmed cases, the number of news articles decreased. In November 2021, the Korean government announced “recovery to a new normal,” easing quarantine policy as vaccination rates increased and public fatigue with social distancing grew [43]. News articles were collected from February 2020 to November 2021, and queries were extracted within this period.

**Figure 3:**
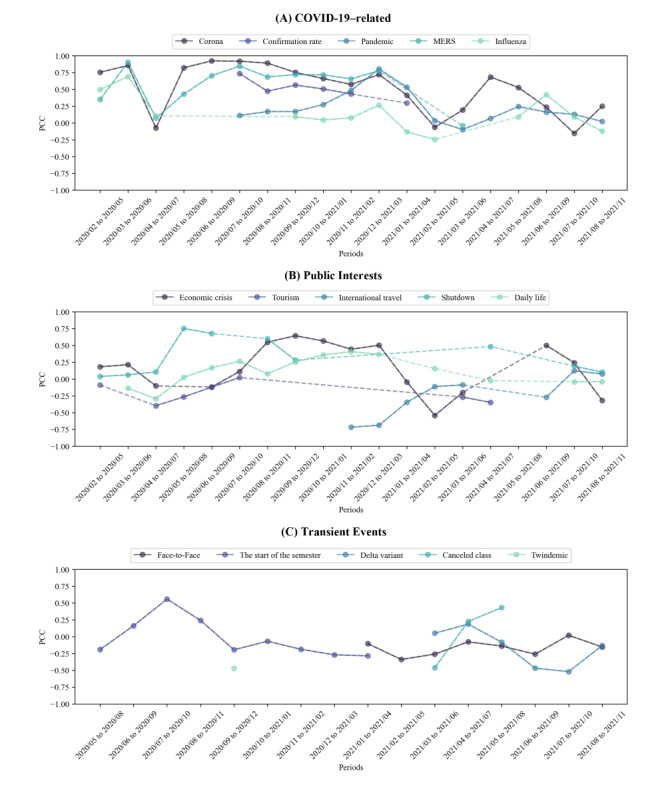
Correlation analysis about extracted queries by query expansion based on the pandemic phase. After categorizing each keyword using expert knowledge, PCC between the number of confirmed cases and (A) keywords directly related to COVID-19, (B) keywords related to public interest, and (C) keywords related to transient events were compared. If a keyword does not appear during the middle period, it is represented by a dotted line. MERS: Middle East respiratory syndrome; PCC: Pearson correlation coefficient.

Next, we extracted word-embedding vectors from news articles over a 4-month time window, sliding this window by 1 month at a time. Subsequently, after removing single-character words, we selected 100-word vectors with the highest cosine similarity with vectors corresponding to “코로나 (Corona)” as keyword queries for further analysis. As a result, 19 sets of 100-word embedding vectors were established between February 2020 and November 2021. For each 4-month interval, we collected search engine query data corresponding to the extracted keywords using the NDL API. We examined the association between the collected search engine queries and the actual number of COVID-19-confirmed cases by calculating the PCC for each period (Figure 3). All extracted keywords and PCC values are presented in Multimedia Appendix 2. Among the expanded queries, words directly related to COVID-19, such as “confirmation rate,” “pandemic,” and “influenza,” exhibited a high PCC in the early stages of the pandemic, but demonstrated a decreasing trend over time (Figure 3A). Past outbreaks of infectious disease, “Middle East respiratory syndrome (MERS),” demonstrated a similar tendency in keywords directly related to COVID-19. Words related to government policy and public interest, such as “shutdown,” “daily life,” “tourism,” “international travel,” and “economic crisis,” partially emerged in response to the decline or resurgence of COVID-19 (Figure 3B). Particularly, the term “economic crisis” exhibited a high positive correlation during periods, including December 2020, when there was a notable increase in COVID-19 cases. Conversely, “international travel” exhibited a negative correlation with the number of confirmed COVID-19 cases during these periods. In Figure 3C, words related to variants or transient events, such as “Delta variant,” “twindemic,” “the beginning of the semester,” “canceled classes,” and “face-to-face” are infrequently observed but show a high correlation when the corresponding events occur.

**Figure 4:**
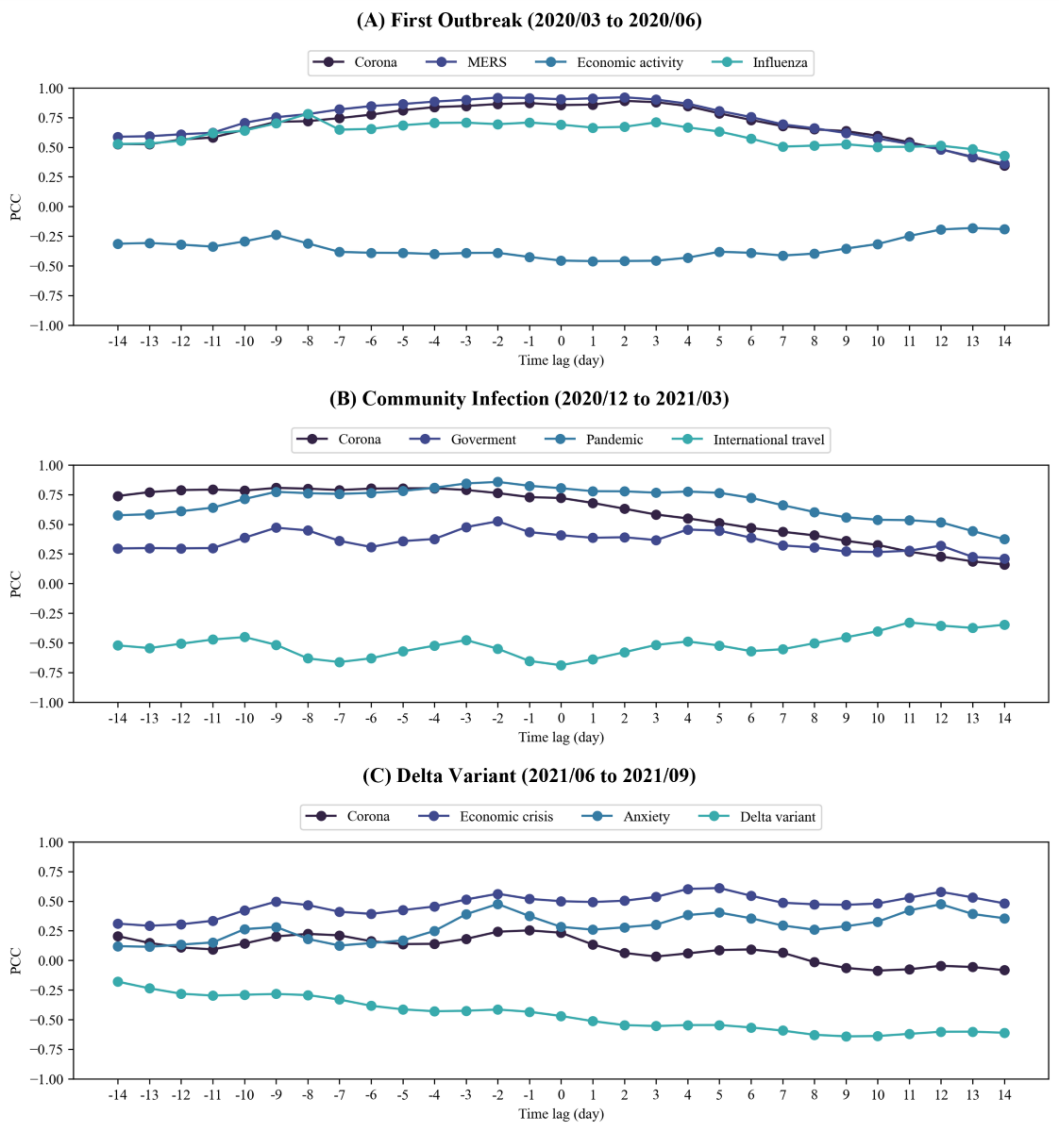
Time-lagged cross-correlation analysis depends on different pandemic phases. Keywords exhibiting high positive or negative PCCs at certain time lag were selected for each phase: (A) the first outbreak, (B) the community infection, and (C) the delta variants. Negative time lags indicate that a query preceded changes in COVID-19–confirmed cases, while positive time lags indicate that a query followed changes in case numbers. PCC: Pearson correlation coefficient.

Given the evidence from previous studies indicating time delay patterns between the number of confirmed COVID-19 cases and search engine queries, we conducted an analysis using TLCC to account for the possibility that search engine queries may precede or follow confirmed cases [25-27]. After shifting the search engine queries within the range of 14 days before to 14 days after, we calculated the PCC of each query based on the number of confirmed cases. The TLCC results are presented in Multimedia Appendix 3. Figure 4 illustrates the TLCC results of distinct keywords during the first outbreak (March 2020 to June 2020), the resurgence of COVID-19 resulting from community infections (December 2020 to March 2021), and the emergence of the delta variant (June 2021 to September 2021). During the initial outbreak, words directly related to “Corona,” such as “MERS,” and “Influenza” exhibited a high correlation with the number of confirmed cases closely aligned with a time lag of zero, consistent with “Corona” (Figure 4A). Conversely, the term “economic activity” exhibited a negative correlation with the number of confirmed cases, reaching its highest absolute value with a time lag of 1, indicating 1 day after. During the resurgence of COVID-19, “corona,” “pandemic,” and “government,” associated with cluster infections and subsequent policies during this period, exhibited a high positive correlation at negative time lags, particularly 9 days before the onset (Figure 4B). Conversely, the PCCs of these terms decreased at positive time lags. Similarly, the term “international travel” displayed a high negative correlation at negative time lags, especially 7 days before the onset. During the period when the delta variants emerged, high positive PCCs of words related to public and social responses, such as “economic crisis” and “anxiety,” were observed at positive time lags, indicating that these search queries preceded the confirmation rate (Figure 4C). The term “Delta variant” showed a high negative correlation at positive time lags. However, the term “corona” displayed low PCCs during this period, differing from the higher relevance observed in previous periods of the first outbreak and community infections.

### Prediction Performance for Daily Confirmed COVID-19 Cases

To evaluate the capability of the query expansion technique to dynamically extract informative keywords as an infectious disease progresses, we proposed a machine learning framework to predict the number of daily confirmed COVID-19 cases using the extracted queries. For each of the 19 four-month period, the first 3 months were used as the training set, whereas the last month was used as the testing set.

To demonstrate that query expansion-based keyword extraction is helpful in predicting daily COVID-19 cases by reflecting the variation in public interest in COVID-19, we compared the prediction performance of symptom-based keywords [18], heuristic-based keywords [17], and past daily confirmed COVID-19 cases only (case-only) [44], which were used in previous studies. Symptom-based keywords for the first few hundred [45] surveys identified 19 symptoms related to daily COVID-19 cases and their frequency of occurrence. Owing to the direct association between COVID-19 and its symptoms, it was used as the basis for symptom-based keywords for COVID-19 in a previous study [18]. To use these keywords, we translated 19 symptoms from the first few hundred into Korean to extract search engine query data from search engines in South Korea. Heuristic-based keywords are COVID-19–related keywords useful for predicting daily COVID-19 cases in South Korea, as selected in a previous correlation study [46]. These keywords also included terms indirectly related to COVID-19 (eg, social distancing and masks). We used the same keywords as in previous studies (Multimedia Appendix 4).

**Figure 5:**
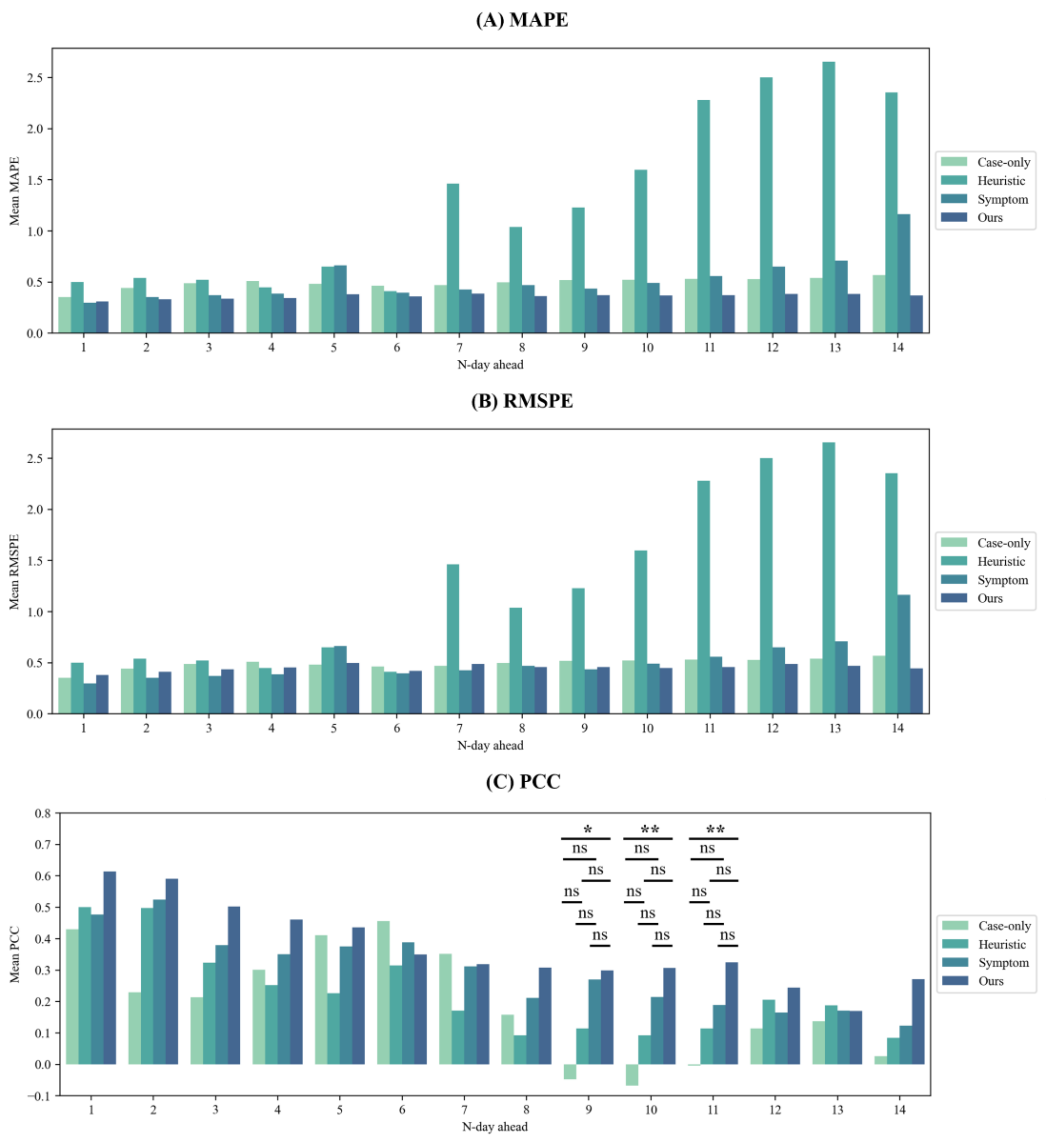
Comparison of prediction performance between our approach, models without queries, and 2 previous query-based methods using experts’ heuristics [17] and symptoms [18]. *P<.05, **P<.01, and ns=not significant. MAPE: mean absolute percentage error; PCC: Pearson correlation coefficient; RMSPE: root-mean-squared percentage error.

In Figure 5 [17,18], we averaged the results of 19 predictions over the entire time period and compared them to previous methods [17,18,44]; the results for each time period are shown in Tables S1, S2, and S3 in Multimedia Appendix 5. We predicted 1-14 days ahead to see how search engine query data extracted by query expansion affects the n-day-ahead prediction. In Figures 5A and 5B [17,18], our method has a lower MAPE than the other methods at 2 and 14 days ahead and a lower RMSPE than the other methods at 8, 10, and 14 days ahead. At 5, 7, and 14 days ahead, the search engine query data for the heuristic method showed a significant increase in MAPE and RMSPE compared with case-only. The MAPE and RMSPE were higher when the search engine query data on the symptom method were 5 days ahead and 11-14 days ahead than case-only. We can see that our method does not change much in MAPE as N-days-ahead increases, has less performance drop than the other methods, and has a lower MAPE and RMSPE than case-only as N-days-ahead increases. Regarding PCC, Figure 5C [17,18] shows that our method outperforms the other methods, except for 6, 7, and 13 days ahead. In particular, at 2, 9, 10, and 11 days ahead, PCC showed significant differences in multiple comparisons using the Kruskal-Wallis H-test (*P*=.04, *P*=.01, *P*<.01, and *P*<.01, respectively). In addition, the PCC showed significant differences between the case-only method and our method at 9, 10, and 11 days ahead of the post-hoc analysis using the Benjamini-Hochberg procedure (*P*=.02, *P*<.01, and *P*<.01). Overall, for most n-day-ahead evaluations, our method showed through MAPE, RMSPE, and PCC that the difference between the predicted and actual values was smaller than that of the other methods, while the predicted values were simultaneously highly correlated with the actual values.

**Figure 6:**
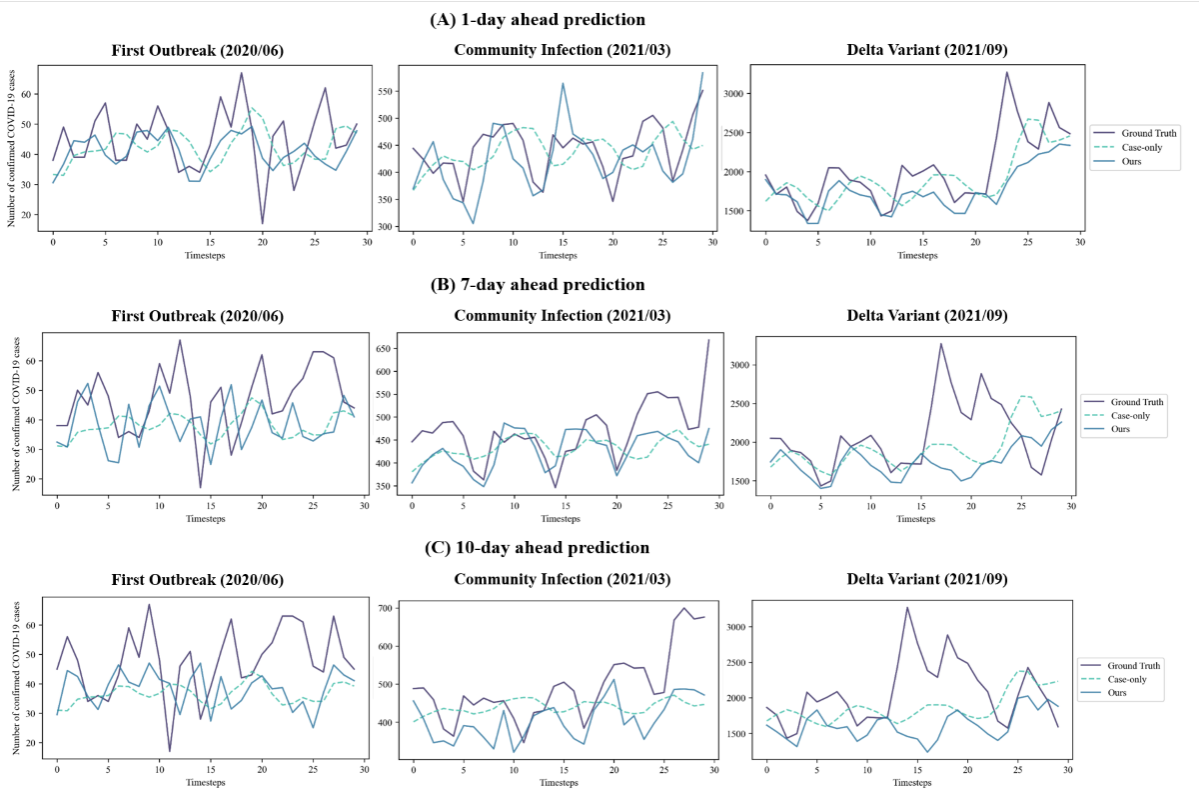
Regression plots on the ground truth values and the predicted values of the case-only method and our method in 1-, 7-, and 10-day ahead prediction.

Figure 6 presents the regression plots comparing the ground truth values and the predicted values from the case-only method and our method. For the 1-day, 7-day, and 10-day ahead predictions, our method demonstrates a time series pattern that is more closely aligned with the ground truth compared with the case-only method. Notably, in the 10-day ahead prediction, where the PCC demonstrates statistical significance, both the case-only method and our method struggle to accurately predict sharp increases in the number of confirmed cases. However, our method more effectively captures the overall pattern of increases and decreases than the case-only method.

To evaluate the effectiveness between the GT data and NDL data, we compared MAPE, RMSPE, and PCC for the 10-day ahead predictions. Additionally, we conducted a post hoc analysis using the Mann-Whitney *U* test with the Benjamini-Hochberg procedure to verify whether the case-only method and other methods based on search engine query data show significant differences in performance. In Table 1, our method using NDL data achieved average MAPE, RMSPE, and PCC values of 0.3654, 0.4462, and 0.3069, respectively, outperforming our method using GT data, which recorded 0.4229, 0.5199, and 0.1918. The post-hoc analysis confirmed that our method using NDL data is the only approach that showed a statistically significant improvement over the case-only method (*P*=.02).

**Table 1:** Prediction performance comparison on Google Trends (GT) data and NAVER Data Lab (NDL) data in 10-day ahead prediction.

Method	Mean absolute percentage error, mean (SD)	Root mean squared percentage error, mean (SD)	Pearson correlation coefficient, mean (SD)	*P* value
Case-only	0.5214 (0.7251)	0.6177 (0.8590)	–0.0678 (0.2990)	—^a^
Heuristic (GT)	0.4824 (0.4581)	0.5738 (0.5711)	0.0664 (0.2891)	.44
Heuristic (NDL)	1.5956 (4.8904)	2.2247 (7.0910)	0.0921 (0.3270)	.44
Symptom (GT)	0.4542 (0.3421)	0.5333 (0.3982)	0.0587 (0.2816)	.44
Symptom (NDL)	0.4907 (0.4518)	0.6868 (0.7460)	0.2142 (0.3362)	.11
Ours (GT)	0.4229 (0.4143)	0.5199 (0.5567)	0.1918 (0.3276)	.15
Ours (NDL)	0.3654 (0.2983)	0.4462 (0.3958)	0.3069 (0.3074)	.02

^a^Not applicable.

## Discussion

### Principal Results

In this study, we proposed a novel approach that uses query expansion techniques to forecast the number of confirmed COVID-19 cases. Specifically, our approach involves automatically extracting search engine queries that are highly relevant to COVID-19 from internet documents. We crawled words that appeared alongside “corona” from news articles and embedded them as word vectors based on their similarity to “corona.” Consequently, the proposed system could effectively adjust to temporal variations in keyword prominence across the various phases of infectious diseases. Internet search behavior patterns vary depending on the prevalence of keywords during specific periods. For example, queries related to symptoms, government policies, public concerns, and social events can emerge at various periods. To reflect these dynamics, queries from particular time intervals that demonstrated the highest TLCC were selected to predict the number of confirmed cases. To the best of our knowledge, this is the first attempt to forecast the number of COVID-19 cases by extracting textual data from web-based documentation using the word embedding method.

In addition, our model demonstrated superior performance compared with those from previous studies [17,18,44]. Furthermore, in predicting confirmed cases 9-11 days after keyword appearance, we exhibited statistically significant superior performance than using only the number of confirmed cases, in contrast to previous studies (*P*=.02, *P*<.01, and *P*<.01; Figure 5C [17,18]). From a medical perspective, the number of COVID-19 cases in the previous period serves as a powerful predictor of future cases [47]. Mathematical models of infectious diseases, such as susceptible-infected-recovered frameworks, have also used a number of confirmed cases as an essential predictor [47]. Moreover, in the initial stage, the number of infected populations was the most important factor, because changes in other factors were minimal. Therefore, we were able to statistically demonstrate that our model, unlike previous models, does not rely solely on the number of confirmed cases for prediction.

Predicting the daily number of confirmed COVID-19 cases can be useful for making decisions related to medical resource allocation and formulating epidemic/pandemic prevention policies [48]. Therefore, it can aid medical and governmental decision-making to prevent the spread of infectious diseases. Conventional studies that forecast the number of patients with infectious diseases have relied on time-series analyses that consider the temporal dynamics of past patient numbers, such as the autoregressive model and LSTM [2,3,8,44]. However, our results demonstrate that the prediction performance of models based solely on past confirmed cases deteriorates for future confirmed cases beyond 7 days (Figure 5C [17,18]). This could arise from external factors such as the emergence of variants, government policies, or social events that may intervene and cause fluctuations in daily COVID-19 cases owing to the virus’ high contagiousness [49].

To address this issue, several studies incorporating search engine queries selected keywords based on researchers’ experiences [17,46] or by using trending COVID-19–related keywords obtained from GTs [12,15,18,41,42], Baidu [16], and NDL [17]. Previous studies have mainly focused on keywords directly related to COVID-19 [17,41,42], keywords associated with COVID-19 symptoms [15,18], and individual preventive materials [17] (Multimedia Appendix 4). These heuristic approaches often functioned effectively, particularly in collaborations with experts. However, this process is time-consuming and resource-intensive, and the results can vary significantly depending on the expertise of the executor. We evaluated the predictive performance of our approach using 2 previously established query sets: symptom-based queries [15,18] and heuristic queries [17]. We identified that heuristic queries deteriorate the prediction performance in most cases compared with models that do not use queries (Figure 5 [17,18]), especially for those beyond 6 days. The symptom-based query resulted in higher PCCs compared with models without a query in all cases, except for 5, 6, and 7 days ahead; however, no statistical difference was found. Additionally, for predictions 11-14 days ahead, the MAPE and RMSPE showed considerable increases, particularly for longer prediction horizons.

We demonstrated that our approach outperforms previous methods in most scenarios. Our method exhibited the lowest MAPE in all N-day-ahead predictions, except for prediction 1-day ahead, where it ranked second following the model with a symptom-based query. Similarly, we also proved the robustness of our approach by observing the highest PCCs in all cases, except for predictions 6 and 7 days ahead, where it placed second, following models without queries. Across all 19 periods, the PCCs between the predicted values and actual outputs were positive, unlike in the other methods, which exhibited negative PCCs in several periods (Figure S3 in Multimedia Appendix 5). In particular, our framework showed the capability to predict confirmed cases more than 1 week in advance, unlike other approaches.

The superior prediction performance of our approach suggests that our framework, which incorporates query expansion techniques and TLCC, adeptly identifies keywords temporally associated with COVID-19 by tracking shifts in public interest regarding the pandemic [26]. Specifically, our approach demonstrated that, beyond keywords directly related to COVID-19 and keywords associated with symptoms or personal protective equipment, a variety of other keywords, including past outbreak infectious diseases such as “MERS” and societal impacts of COVID-19 such as “economic crisis,” “the start of the semester,” and “international travel,” could also be effectively used to predict COVID-19 cases (Figure 3). These keywords suggest that using not only articles directly related to COVID-19 but also non–COVID-related or indirectly related articles can aid in extracting valuable keywords. Interestingly, MERS, a past infectious disease outbreak similar to COVID-19, exhibited a comparable trend of a high positive correlation during this stage. This result stemmed from numerous media articles that drew parallels and distinctions between COVID-19 and MERS.

To evaluate the generalization of our method in other infectious diseases, we applied the query expansion approach to MERS-CoV-2 (MERS) outbreak, which occurred in South Korea in 2015. After crawling news articles using the keyword “MERS” from the 2-month outbreak period, spanning May 20 (ie, the date of the first confirmed case) to July 19 (ie, the release date of the Intensive Care Hospital) in 2015, we analyzed the correlation between MERS-related queries and the number of cases. To evaluate the effectiveness of query expansion, we compared the correlation coefficients of our query set with those from previous studies [50,51], as presented in Multimedia Appendix 6. When comparing the top 3 keywords in terms of PCC, our method extracted “확진” (“confirmation,” PCC=0.6533), “환자” (“patient,” PCC=0.6472), and “감염” (“infection,” PCC=0.5784). In contrast, the heuristic method extracted “메르스” (“MERS,” PCC=0.5708), “MERS” (PCC=0.5478), and “메르스병원” (“MERS hospital,” PCC=0.5176), while the symptom-based method extracted “기침” (“cough,” PCC=0.3956), “설사” (“diarrhea,” PCC=0.2687), and “두통” (“headache,” PCC=0.2663). The results indicate that our method successfully extracted keywords with stronger correlations, demonstrating its potential for application across various infectious disease outbreaks to obtain search engine query data significantly correlated with confirmed case trends, thereby enhancing predictive performance. However, the MERS outbreak was much shorter than the COVID-19 pandemic, lasting approximately 2 months, with a peak in reported cases in early June. Developing predictive models for this epidemic is challenging due to the limited time-series data available, particularly for the period preceding the June outbreak. A primary limitation of our query expansion framework is its dependence on a substantial amount of textual data. The amount of text data from May 20 to early June may be insufficient to perform effective word embedding. The objective of correlation analysis was to determine whether the keywords generated through query expansion significantly correlated with the actual case counts, thereby assessing the effectiveness of our method in extracting relevant search queries under limited data conditions.

In addition, we discovered that keywords exhibiting a negative TLCC could also be effectively used to predict COVID-19 cases (Figures 3 and 4). Keywords such as “economic activity” and “international travel” may reflect this negative correlation due to a shift in public focus toward daily life as incidences of COVID-19 decline. The keyword “Delta variant” also showed a negative correlation, which could be attributed to the extensive attention it received prior to its spread in South Korea. However, once present in South Korea, the epidemiological characteristics did not significantly differ from those of previous variants [52,53], which may explain the observed negative correlation. For the MERS outbreak, we discovered keywords related to the MERS, quarantine, and infected facilities using TLCC analysis. The MERS-related keywords included “메르스” (“MERS”), “감염” (“Infection”), “전염” (“Contagion”), “환자” (“Patient”), “확진” (“Confirmation”), and “사망자” (“Dead”). Keywords related to quarantine were “격리” (“Isolation”), “보건복지부” (“Ministry of Health and Welfare”), and “정부” (“Government”). For infected facilities, “병원” (“Hospital”) and “평택” (“Pyeongtaek”) were extracted. Among the MERS-related keywords, “메르스” (“MERS”), “감염” (“Infection”), and “전염” (“Contagion”), as well as those related to quarantine and infected facilities, showed the highest correlation within a time lag range of -3 to -5 days, suggesting that these keywords reflect public interest prior to the confirmation of cases. In contrast, “환자” (“Patient”), “확진” (“Confirmation”), and “사망자” (“Dead”) exhibited the highest correlations at time lags of 1, 2, and 4 days, respectively, indicating that these keywords capture increased public interest following case confirmations. Detailed TLCC results for MERS are provided in Multimedia Appendix 7.

We discovered changes in public interest toward the pandemic over time. During the first outbreak, there was a high positive correlation observed among keywords directly linked to COVID-19, such as “Corona,” “Influenza,” and “Confirmation rate” (Figures 3 and 4). This trend was likely a reflection of intensified public attention toward COVID-19 as a result of frequent search queries related to confirmed case counts. The PCCs of these keywords exhibited a declining trend over time, suggesting a potential decrease in public interest in the number of confirmed COVID-19 cases. In addition, by analyzing the TLCC during the first outbreak, most keywords were aligned with no time lag. During this period, the government accurately tracked all confirmed cases, and people’s interest in COVID-19 was exceptionally high. Consequently, it can be hypothesized that there is a correlation between the frequency of keywords appearing in news articles and the number of COVID-19 cases, which may be closely aligned in real time.

In the community infection period, because the government started to fail to track all routes of confirmed cases, keywords regarding the government’s pandemic control policies, such as “Shutdown” and “Daily life,” were frequently observed with high positive correlations, alongside keywords directly associated with COVID-19 (Figure 3B). This observation suggests that increased public interest in government pandemic control policies could be a contributing factor. Interestingly, the appearance of COVID-19-related keywords tends to align well with the confirmed case numbers 7-14 days after their appearance, as indicated by the peaks in the negative time lags (Figure 4). It was hypothesized that an increase in COVID-19 cases impacts daily life, leading to an increase in keywords in news articles before individuals receive a confirmed diagnosis. Thus, during this period, an internet search–based artificial intelligence model could significantly aid in predicting COVID-19 cases. Consequently, our approach exhibited a superior prediction performance during these periods, particularly with the highest number of positive PCCs (Figure S3 in Multimedia Appendix 5).

During the delta variant period, there was a general decrease in the PCC between keyword frequency in news articles and the number of confirmed cases. Specifically, keywords directly related to COVID-19, including “Corona,” showed relatively low TLCC in all time lags. Instead, keywords related to the societal impacts of COVID-19, such as “economic crisis” and “anxiety,” exhibited high TLCCs in positive time lags. This indicates a tendency for these keywords to appear in news articles following an increase in confirmed cases. The patterns during this period suggest that public interest in COVID-19 diminished overall, and even in cases of symptoms or confirmed infections, the issue was not treated with urgency or importance. Because the prediction of confirmed cases should be performed based on previous search engine queries (ie, TLCCs at negative time lags), our approach demonstrated relatively low PCCs during these periods, despite showing comparably low MAPE and RMSPE values (Figure S3 in Multimedia Appendix 5).

### Limitations

Despite the remarkable predictive performance of our approach, particularly for predictions beyond 1 week ahead, our study has several limitations. First, we collected and used a corpus of news articles to ensure data reliability. During the initial stages, when there was significant interest in the pandemic, the number of news searches and confirmed cases were relatively well correlated. However, as time progressed and interest in the disease declined, the predictive capacity decreased. Further research incorporating a corpus that can effectively predict confirmed cases even when interest in news searches declines may hold substantial potential for improvement.

Second, owing to the reliance on news articles for data collection, the total amount of data yielded was relatively small. Consequently, we were limited to training Word2Vec on 4 months’ worth of data for COVID-19 outbreak. This limitation could potentially be addressed by collecting additional, validated textual data on a large scale, or by devising methodologies that can enable word embedding or keyword extraction even with minimal data. From a methodological perspective on word embedding, FastText was found to be less suitable compared with Word2Vec-CBOW, as it tends to focus more on morphological features rather than the semantic aspects of words. Additionally, Word2Vec-Skipgram, which requires a larger number of training iterations and more data than Word2Vec-CBOW, demonstrated a tendency to extract less relevant keywords when trained under the same parameter settings and dataset, making it comparatively less effective. Overcoming this limitation may allow the extraction of keywords within a narrower temporal range, thereby enabling a more sensitive capture of shifts in public interest toward infectious diseases.

### Conclusions

In this study, a COVID-19 case prediction model was developed using web-based search data and the Word2Vec algorithm with adaptability to temporal variations. This was achieved through direct text extraction from internet-based documents. The model outperformed previous models, even with automated keyword selection and without expert experience. Additionally, analysis of the keywords used provided insights into shifts in the public interest, correlating with changes in case numbers. This study highlighted the potential utility of using machine learning and search data to track and predict disease progression.

## Appendix

Multimedia Appendix 1 Basic information of the collected text data from news articles.

Multimedia Appendix 2 Extracted query lists using query expansion per period.

Multimedia Appendix 3 Time-lagged cross-correlation (TLCC) values and optimal time lag on all periods and queries.

Multimedia Appendix 4 Query lists about heuristics and symptoms in previous studies.

Multimedia Appendix 5 Performance tables and figures about averaged n-day ahead and averaged periods.

Multimedia Appendix 6 Pearson correlation coefficients of Middle East respiratory syndrome (MERS)–related queries.

Multimedia Appendix 7 Time-lagged cross-correlation (TLCC) analysis of Middle East respiratory syndrome (MERS)–related queries.

## Abbreviations

API: application programming interface
GT: Google Trends
MAE: mean absolute error
MAPE: mean absolute percentage error
MERS: Middle East respiratory syndrome
NDL: NAVER Data Lab
PCC: Pearson correlation coefficient
RMSE: root-mean-squared error
RMSPE: root-mean-squared percentage error
TLCC: time-lagged cross-correlation
