# Supplementary material for: Discovering Time-Varying Public Interest for COVID-19 Case Prediction in South Korea Using Search Engine Queries: Infodemiology Study
Source: J Med Internet Res. 2024 Dec 16;26:e63476. doi: 10.2196/63476 (PMC11686031; doi:10.2196/63476)

Table S1. MAPE Performance on averaged periods

| N-day ahead | Case-only | Heuristic | Symptom | Ours |
| --- | --- | --- | --- | --- |
| 1 | 0.3501±0.6077 | 0.4978±0.7693 | **0.2948±0.1937** | 0.3082±0.3258 |
| 2 | 0.4402±0.8212 | 0.5380±0.8239 | 0.3517±0.3381 | **0.3306±0.3336** |
| 3 | 0.4863±0.9643 | 0.5187±0.7604 | 0.3688±0.3823 | **0.3348±0.3364** |
| 4 | 0.5082±1.0707 | 0.4478±0.5705 | 0.3854±0.3498 | **0.3420±0.3262** |
| 5 | 0.4797±0.9340 | 0.6492±1.0995 | 0.6615±1.3936 | **0.3778±0.4240** |
| 6 | 0.4612±0.8222 | 0.4079±0.3779 | 0.3928±0.3432 | **0.3558±0.2939** |
| 7 | 0.4668±0.7625 | 1.4592±4.5156 | 0.4250±0.4012 | **0.3859±0.3811** |
| 8 | 0.4967±0.7329 | 1.0379±2.5349 | 0.4689±0.2924 | **0.3587±0.3061** |
| 9 | 0.5183±0.7300 | 1.2263±3.2982 | 0.4352±0.3053 | **0.3680±0.2949** |
| 10 | 0.5214±0.7251 | 1.5956±4.8904 | 0.4907±0.4518 | **0.3654±0.2983** |
| 11 | 0.5281±0.7140 | 2.2786±7.8131 | 0.5565±0.7121 | **0.3695±0.2891** |
| 12 | 0.5249±0.7007 | 2.5011±8.8636 | 0.6479±1.1718 | **0.3806±0.2908** |
| 13 | 0.5382±0.6913 | 2.6526±9.4078 | 0.7086±1.4015 | **0.3831±0.2407** |
| 14 | 0.5658±0.6872 | 2.3525±8.2029 | 1.1638±3.1119 | **0.3674±0.2191** |

Table S2. RMSPE Performance on averaged periods

| N-day ahead | Case-only | Heuristic | Symptom | Ours |
| --- | --- | --- | --- | --- |
| 1 | 0.5258±1.1118 | 0.7813±1.3481 | 0.4199±0.3624 | **0.3798±0.4237** |
| 2 | 0.6686±1.5140 | 0.8120±1.4821 | 0.4635±0.5722 | **0.4108±0.4588** |
| 3 | 0.7592±1.8322 | 0.6644±1.1014 | 0.4722±0.5625 | **0.4345±0.5191** |
| 4 | 0.8139±2.0807 | 0.5930±0.8421 | 0.5094±0.5425 | **0.4517±0.5791** |
| 5 | 0.6385±1.3576 | 0.8842±1.6623 | 0.9563±2.0084 | **0.4950±0.6965** |
| 6 | 0.5450±0.9582 | 0.5187±0.5393 | 0.6010±0.8399 | **0.4194±0.3351** |
| 7 | 0.5551±0.8894 | 1.9675±6.2124 | 0.6540±1.1073 | **0.4849±0.5451** |
| 8 | 0.5869±0.8609 | 1.4104±3.6806 | 0.7319±0.8056 | **0.4554±0.4480** |
| 9 | 0.6116±0.8593 | 1.7397±4.9602 | 0.5144±0.3753 | **0.4552±0.4109** |
| 10 | 0.6177±0.8590 | 2.2247±7.0910 | 0.6868±0.7460 | **0.4462±0.3958** |
| 11 | 0.6322±0.8532 | 3.1967±11.2963 | 0.8651±1.5467 | **0.4553±0.3986** |
| 12 | 0.6342±0.8497 | 3.6475±13.3563 | 0.9173±1.9462 | **0.4865±0.4430** |
| 13 | 0.6472±0.8474 | 3.9708±14.5246 | 1.0360±2.4832 | **0.4677±0.3063** |
| 14 | 0.6752±0.8464 | 3.4410±12.4546 | 1.5682±4.5141 | **0.4420±0.2541** |

Table S3. PCC Performance on averaged periods

| N-day ahead | Case-only | Heuristic | Symptom | Ours |
| --- | --- | --- | --- | --- |
| 1 | 0.4294±0.3346 | 0.5005±0.3189 | 0.4760±0.3346 | **0.6130±0.2833** |
| 2 | 0.2291±0.4205 | 0.4975±0.3355 | 0.5243±0.2700 | **0.5901±0.3082** |
| 3 | 0.2134±0.4033 | 0.3235±0.4096 | 0.3794±0.2924 | **0.5017±0.3362** |
| 4 | 0.3001±0.3507 | 0.2519±0.3627 | 0.3504±0.3298 | **0.4606±0.3692** |
| 5 | 0.4111±0.3039 | 0.2259±0.4048 | 0.3753±0.3438 | **0.4351±0.3351** |
| 6 | **0.4555±0.2761** | 0.3148±0.3855 | 0.3876±0.3086 | 0.3492±0.3076 |
| 7 | **0.3516±0.2710** | 0.1703±0.3423 | 0.3119±0.2730 | 0.3189±0.2791 |
| 8 | 0.1577±0.3576 | 0.0917±0.3569 | 0.2114±0.2786 | **0.3073±0.2614** |
| 9 | -0.0485±0.4157 | 0.1137±0.3584 | 0.2699±0.3553 | **0.2983±0.2748** |
| 10 | -0.0678±0.2990 | 0.0921±0.3270 | 0.2142±0.3362 | **0.3069±0.3074** |
| 11 | -0.0044±0.2450 | 0.1140±0.3128 | 0.1887±0.3350 | **0.3248±0.2560** |
| 12 | 0.1141±0.2749 | 0.2047±0.3650 | 0.1643±0.2979 | **0.2440±0.2417** |
| 13 | 0.1367±0.3593 | **0.1876±0.4035** | 0.1704±0.3182 | 0.1692±0.3578 |
| 14 | 0.0254±0.3573 | 0.0835±0.4057 | 0.1227±0.3212 | **0.2706±0.3425** |

Table S4. MAPE Performance on averaged n-day ahead

| Window | Case-only | Heuristic | Symptom | Ours |
| --- | --- | --- | --- | --- |
| 2020/02~2020/05 | 3.7599±0.5419 | 16.933±15.4758 | 3.679±3.495 | **1.463±0.311** |
| 2020/03~2020/06 | **0.2546±0.0198** | 0.3462±0.1128 | 0.4252±0.0927 | 0.27±0.0291 |
| 2020/04~2020/07 | **0.311±0.014** | 2.0987±0.8978 | 0.4002±0.052 | 0.4109±0.0703 |
| 2020/05~2020/08 | **0.5284±0.1032** | 0.7227±0.0907 | 0.7393±0.1313 | 0.739±0.0864 |
| 2020/06~2020/09 | 0.4596±0.0724 | 0.4267±0.1408 | 0.533±0.1512 | **0.3263±0.078** |
| 2020/07~2020/10 | **0.2815±0.0232** | 0.3996±0.0611 | 0.4469±0.0521 | 0.393±0.0563 |
| 2020/08~2020/11 | **0.4777±0.1438** | 0.5984±0.1063 | 0.5307±0.1205 | 0.523±0.1236 |
| 2020/09~2020/12 | 0.4026±0.2574 | 0.3804±0.2635 | 0.338±0.2102 | **0.2752±0.1225** |
| 2020/10~2021/01 | 0.1316±0.0294 | 0.1288±0.0276 | 0.1627±0.0971 | **0.1223±0.0345** |
| 2020/11~2021/02 | 0.2839±0.0679 | **0.1627±0.0142** | 0.2118±0.0596 | 0.203±0.0394 |
| 2020/12~2021/03 | 0.3893±0.1156 | 0.2824±0.0912 | 0.2535±0.0639 | **0.1968±0.0401** |
| 2021/01~2021/04 | 0.2293±0.0451 | 0.2598±0.0547 | 0.2478±0.0555 | 0.2123±0.075 |
| 2021/02~2021/05 | 0.1347±0.0096 | 0.1391±0.0212 | 0.1392±0.0192 | **0.129±0.0319** |
| 2021/03~2021/06 | 0.2404±0.0511 | 0.2347±0.0521 | **0.2343±0.0673** | 0.2079±0.0574 |
| 2021/04~2021/07 | 0.4863±0.0915 | 0.5075±0.121 | 0.5078±0.0808 | **0.4018±0.0946** |
| 2021/05~2021/08 | 0.1601±0.0283 | 0.1388±0.0284 | 0.2722±0.1708 | **0.12±0.0268** |
| 2021/06~2021/09 | 0.194±0.0329 | 0.2144±0.0288 | **0.1795±0.0429** | 0.1879±0.0304 |
| 2021/07~2021/10 | **0.23±0.0225** | 0.2838±0.0534 | 0.2973±0.0576 | 0.2504±0.0396 |
| 2021/08~2021/11 | 0.3902±0.1015 | 0.3924±0.0647 | **0.3789±0.115** | 0.3917±0.0859 |

Table S5. RMSPE Performance on averaged n-day ahead

| Window | Case-only | Heuristic | Symptom | Ours |
| --- | --- | --- | --- | --- |
| 2020/02~2020/05 | 5.342±1.7724 | 25.2913±23.12 | 6.1089±5.0423 | **2.1507±0.5606** |
| 2020/03~2020/06 | **0.3638±0.0394** | 0.4994±0.1899 | 0.4779±0.0957 | 0.3753±0.0341 |
| 2020/04~2020/07 | **0.4249±0.0358** | 3.0648±1.4234 | 0.5441±0.0733 | 0.5839±0.0837 |
| 2020/05~2020/08 | **0.5842±0.0977** | 0.7543±0.075 | 0.774±0.1247 | 0.7762±0.0653 |
| 2020/06~2020/09 | 0.6218±0.0928 | 0.5892±0.1853 | 0.7218±0.1972 | **0.431±0.1014** |
| 2020/07~2020/10 | **0.3979±0.0457** | 0.574±0.2186 | 0.6161±0.0592 | 0.5298±0.0817 |
| 2020/08~2020/11 | **0.5117±0.1374** | 0.6196±0.1017 | 0.5555±0.1125 | 0.5494±0.1179 |
| 2020/09~2020/12 | 0.5388±0.3479 | 0.502±0.3562 | 0.4516±0.2922 | **0.3513±0.1667** |
| 2020/10~2021/01 | 0.1672±0.0356 | 0.1605±0.0351 | 0.2016±0.1113 | **0.1472±0.0396** |
| 2020/11~2021/02 | 0.3397±0.0684 | **0.2027±0.0158** | 0.2535±0.0669 | 0.2331±0.0381 |
| 2020/12~2021/03 | 0.4615±0.1234 | 0.3445±0.1056 | 0.2992±0.0679 | **0.234±0.0431** |
| 2021/01~2021/04 | 0.253±0.0428 | 0.2788±0.0487 | 0.2656±0.0545 | **0.2254±0.071** |
| 2021/02~2021/05 | **0.1603±0.0092** | 0.1754±0.0283 | 0.1661±0.0232 | 0.1652±0.0419 |
| 2021/03~2021/06 | 0.285±0.0569 | 0.2804±0.0608 | 0.2764±0.0768 | **0.2423±0.0695** |
| 2021/04~2021/07 | 0.495±0.0886 | 0.5169±0.119 | 0.5171±0.0793 | **0.413±0.0911** |
| 2021/05~2021/08 | 0.1933±0.0298 | 0.1739±0.0374 | 0.8705±0.8367 | **0.1392±0.0249** |
| 2021/06~2021/09 | 0.2469±0.0463 | 0.2693±0.0428 | **0.2253±0.0526** | 0.2337±0.0333 |
| 2021/07~2021/10 | **0.2833±0.0274** | 0.3597±0.0629 | 0.367±0.062 | 0.3173±0.0425 |
| 2021/08~2021/11 | 0.4236±0.1009 | 0.4282±0.0648 | **0.4169±0.111** | 0.4307±0.085 |

Table S6. PCC Performance on averaged n-day ahead

| Window | Case-only | Heuristic | Symptom | Ours |
| --- | --- | --- | --- | --- |
| 2020/02~2020/05 | 0.1264±0.1767 | -0.3414±0.2897 | **0.354±0.1133** | 0.0609±0.2022 |
| 2020/03~2020/06 | 0.0038±0.1649 | 0.0852±0.2411 | -0.065±0.1525 | **0.114±0.1317** |
| 2020/04~2020/07 | -0.0365±0.1034 | -0.1065±0.1977 | -0.0426±0.1291 | **0.1458±0.1241** |
| 2020/05~2020/08 | 0.1889±0.5703 | 0.1498±0.4344 | **0.2438±0.3514** | 0.0694±0.4418 |
| 2020/06~2020/09 | **0.6699±0.0889** | 0.6419±0.1678 | 0.5391±0.2315 | 0.5039±0.2247 |
| 2020/07~2020/10 | **0.3507±0.1619** | -0.1311±0.1354 | 0.1484±0.2001 | 0.0352±0.1563 |
| 2020/08~2020/11 | 0.5615±0.4194 | 0.6078±0.2828 | **0.6386±0.3375** | 0.5987±0.4472 |
| 2020/09~2020/12 | 0.1458±0.4805 | 0.1666±0.4724 | 0.1613±0.4734 | **0.2138±0.4525** |
| 2020/10~2021/01 | 0.2068±0.228 | 0.2682±0.1888 | 0.5226±0.1746 | **0.6332±0.0827** |
| 2020/11~2021/02 | -0.1033±0.2483 | 0.0085±0.2747 | 0.0648±0.2392 | **0.2799±0.0915** |
| 2020/12~2021/03 | 0.6156±0.1631 | 0.5819±0.2045 | 0.6121±0.2449 | **0.6191±0.202** |
| 2021/01~2021/04 | 0.04±0.1589 | 0.254±0.2835 | 0.2299±0.3337 | **0.7313±0.0917** |
| 2021/02~2021/05 | -0.006±0.2293 | 0.2627±0.194 | 0.3221±0.2367 | **0.5103±0.1049** |
| 2021/03~2021/06 | 0.1897±0.1527 | 0.2683±0.2987 | 0.3697±0.2742 | **0.4532±0.2074** |
| 2021/04~2021/07 | 0.2603±0.3201 | 0.3243±0.2786 | 0.2753±0.2975 | **0.549±0.2143** |
| 2021/05~2021/08 | -0.0067±0.4193 | 0.2412±0.3243 | 0.1359±0.2503 | **0.6487±0.1001** |
| 2021/06~2021/09 | 0.0617±0.2567 | 0.0464±0.3604 | **0.2344±0.3127** | 0.1693±0.2629 |
| 2021/07~2021/10 | -0.0031±0.3235 | 0.496±0.2648 | **0.5234±0.1671** | 0.362±0.1719 |
| 2021/08~2021/11 | 0.4035±0.5002 | **0.4809±0.38** | 0.36±0.3342 | 0.3454±0.3326 |

Figure S1. MAPE Performance on averaged n-day ahead


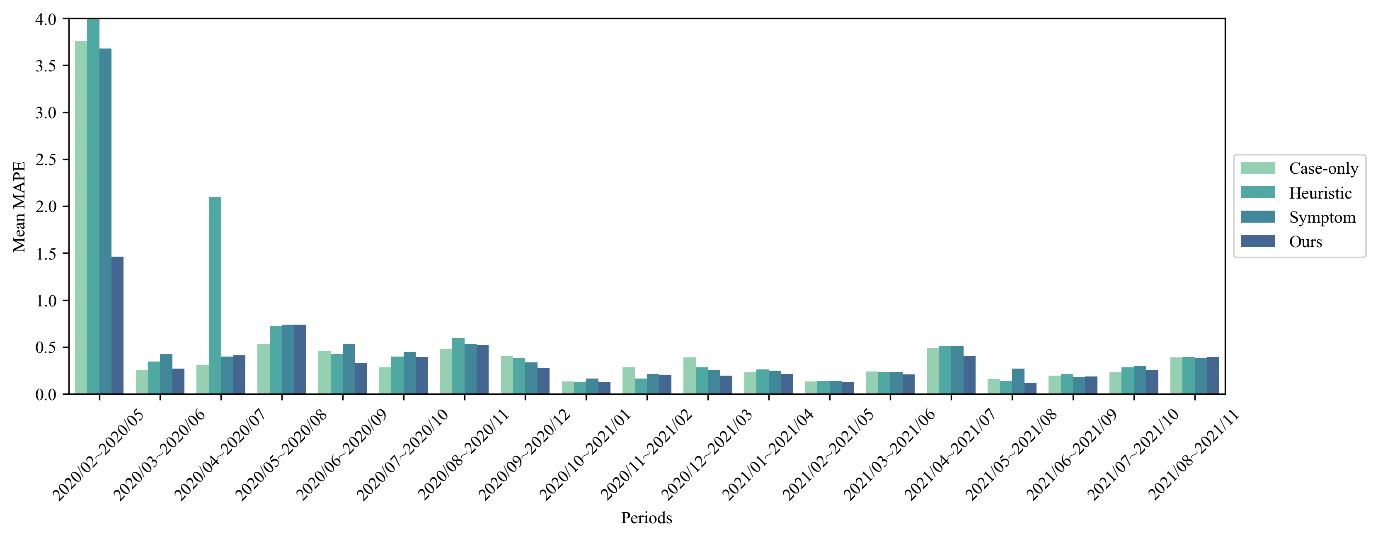


Figure S2. RMSPE Performance on averaged n-day ahead


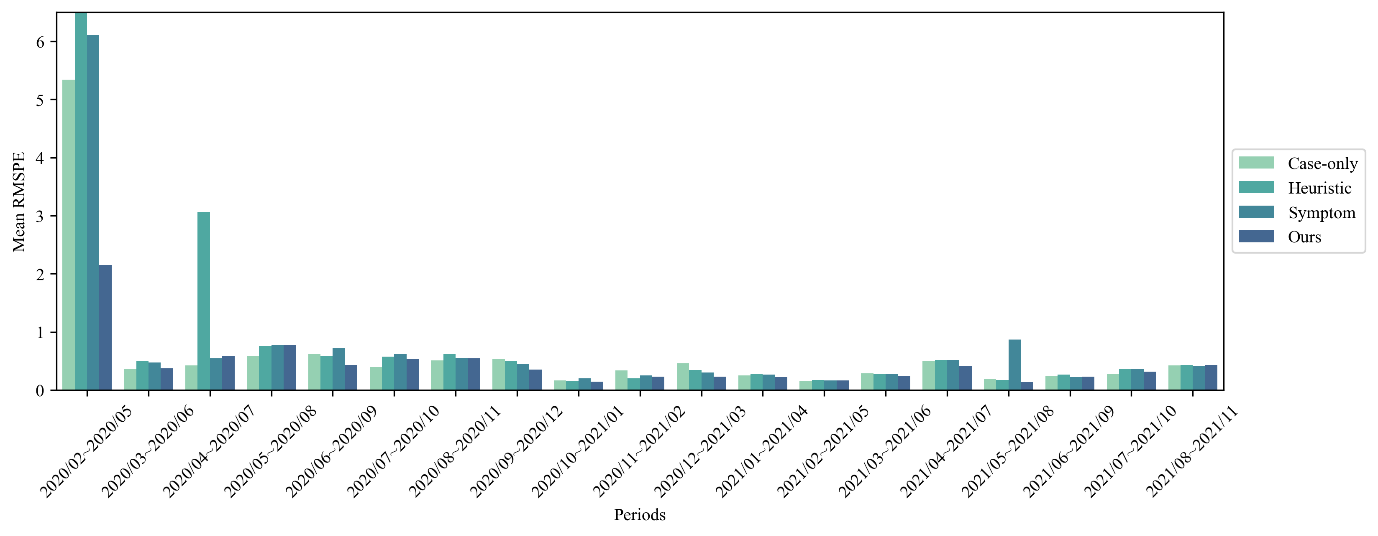


Figure S3. PCC Performance on averaged n-day ahead


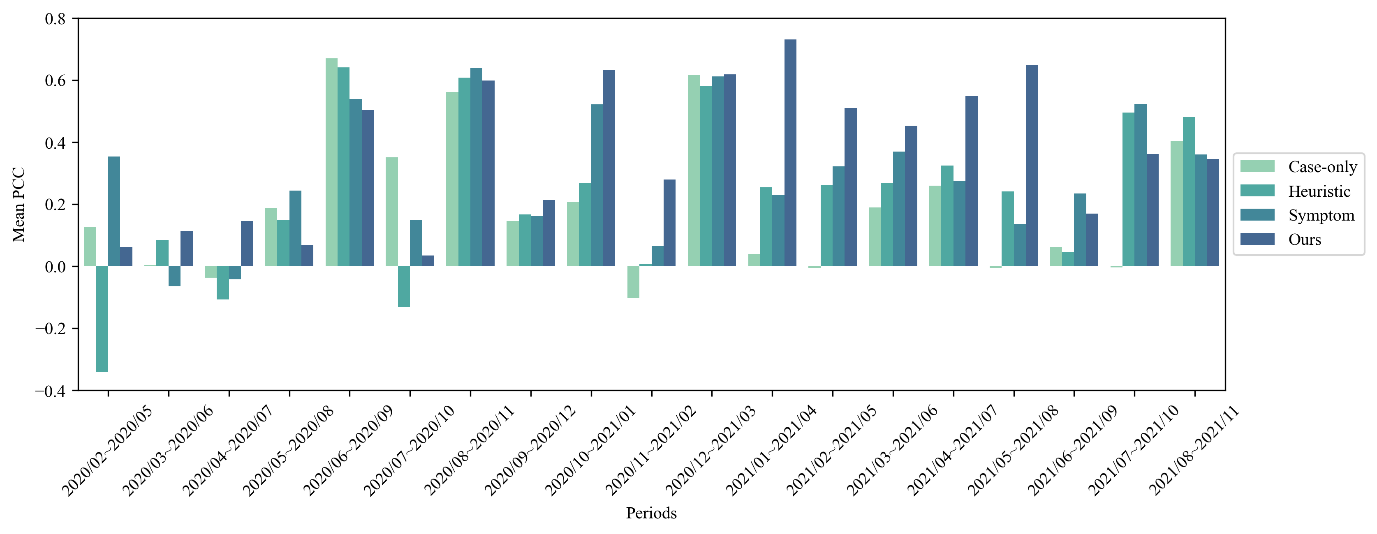

Supplement: Multimedia Appendix 5 [file jmir_v26i1e63476_app5.docx]
